# DRGNet: Enhanced VVC Reconstructed Frames Using Dual-Path Residual Gating for High-Resolution Video

**DOI:** 10.3390/s25123744

**Published:** 2025-06-15

**Authors:** Zezhen Gai, Tanni Das, Kiho Choi

**Affiliations:** 1 Department of Electronics and Information Convergence Engineering, Kyung Hee University, Yongin 17104, Republic of Korea; gaizezhen@khu.ac.kr (Z.G.); tannidas@khu.ac.kr (T.D.); 2 Department of Electronic Engineering, Kyung Hee University, Yongin 17104, Republic of Korea

**Keywords:** post-processing, residual network, CNN, video denoising, high-resolution video

## Abstract

In recent years, with the rapid development of the Internet and mobile devices, the high-resolution video industry has ushered in a booming golden era, making video content the primary driver of Internet traffic. This trend has spurred continuous innovation in efficient video coding technologies, such as Advanced Video Coding/H.264 (AVC), High Efficiency Video Coding/H.265 (HEVC), and Versatile Video Coding/H.266 (VVC), which significantly improves compression efficiency while maintaining high video quality. However, during the encoding process, compression artifacts and the loss of visual details remain unavoidable challenges, particularly in high-resolution video processing, where the massive amount of image data tends to introduce more artifacts and noise, ultimately affecting the user’s viewing experience. Therefore, effectively reducing artifacts, removing noise, and minimizing detail loss have become critical issues in enhancing video quality. To address these challenges, this paper proposes a post-processing method based on Convolutional Neural Network (CNN) that improves the quality of VVC-reconstructed frames through deep feature extraction and fusion. The proposed method is built upon a high-resolution dual-path residual gating system, which integrates deep features from different convolutional layers and introduces convolutional blocks equipped with gating mechanisms. By ingeniously combining gating operations with residual connections, the proposed approach ensures smooth gradient flow while enhancing feature selection capabilities. It selectively preserves critical information while effectively removing artifacts. Furthermore, the introduction of residual connections reinforces the retention of original details, achieving high-quality image restoration. Under the same bitrate conditions, the proposed method significantly improves the Peak Signal-to-Noise Ratio (PSNR) value, thereby optimizing video coding quality and providing users with a clearer and more detailed visual experience. Extensive experimental results demonstrate that the proposed method achieves outstanding performance across Random Access (RA), Low Delay B-frame (LDB), and All Intra (AI) configurations, achieving BD-Rate improvements of 6.1%, 7.36%, and 7.1% for the luma component, respectively, due to the remarkable PSNR enhancement.

## 1. Introduction

In recent years, with the widespread adoption of mobile devices and the rapid development of the Internet, video playback data has experienced explosive growth. Short videos, with their engaging content and diverse formats, have been deeply integrated into various aspects of daily life. Alongside technological advancements and consumption upgrades, the demand for high-resolution videos has also been increasing rapidly. However, the rapid growth of the video industry has brought significant challenges, such as the increasing bandwidth requirements for video transmission, imposing substantial cost pressures on many video platforms. This burden directly impacts video playback quality, hindering the delivery of meticulously crafted high-quality content in its original form to viewers. This issue is particularly pronounced in high-resolution playback scenarios, where viewing experiences can noticeably deteriorate. Such limitations not only dampen the enthusiasm of video creators but over time, may also reduce audience interest and engagement with video content. In this context, video coding standards have played a crucial role in managing the surge in video traffic by improving compression efficiency and reducing transmission costs.

### 1.1. Signal Processing-Based Filtering Techniques

In video coding, traditional video encoding is typically based on a block-based hybrid video coding framework. Within this structure, each individual coding tool exhibits different levels of coding efficiency. This method has been widely applied in various video coding standards, such as AVC [1], HEVC [2], and VVC [3]. Before entering the encoding process, the input video sequence is first processed and divided into blocks. It then goes through a prediction module, which includes intra-frame prediction using pixel information from the current frame and inter-frame prediction based on motion compensation from reference frames. The system then calculates the difference between the predicted value and the actual value, known as the residual, which is subsequently transformed and quantized. Finally, the quantized coefficients undergo entropy coding to generate the final bitstream.

In the decoding loop, the decoder performs inverse quantization and inverse transformation to reconstruct the residual, providing a reference for inter-frame prediction. The decoded image then passes through a series of loop filtering modules, including Adaptive Loop Filtering (ALF) [4], Sample Adaptive Offset (SAO) [5,6], Deblocking Filter (DBF) [7,8], and Luminance Mapping with Chroma Scaling (LMCS) [9,10]. After processing through these filters, the filtered decoded image is stored in the reference frame buffer for subsequent inter-frame prediction. Ultimately, this process produces a compressed bitstream compliant with the standard as the final output.

Signal processing-based filters in the video coding standards have various limitations. For example, although ALF enhances texture details by analyzing block characteristics and designing adaptive filter coefficients, it significantly increases computational complexity and may introduce excessive smoothing, leading to insufficient edge sharpening. SAO has limited local adaptive capability and relies too much on classification rules, making it difficult to capture complex image features, resulting in blurry edge textures and reduced overall performance. DBF, while reducing block artifacts, may cause over-smoothing under strong filtering conditions, damaging texture and edge detail performance. Additionally, in dynamic scenes or high-noise images, it may amplify background noise and texture interference. LMCS is constrained by global mapping, lacking adaptive processing for local areas. In high dynamic range (HDR) or high-contrast scenes, this can lead to loss of detail and potential color distortion, placing higher demands on decoding performance.

### 1.2. Neural Network-Based Filtering Techniques

To overcome the limitations of traditional signal processing-based filters, researchers have increasingly focused on CNN-based loop filters. By learning complex non-linear mapping relationships, CNN can dynamically adjust filtering strategies according to different scenes. Through training on diverse image datasets, CNN can efficiently learn how to handle various distortions, such as chromatic aberration, noise, and compression artifacts [11,12,13,14,15], significantly improving their robustness and image restoration capabilities, especially when dealing with unknown datasets. A richer dataset provides CNNs with more samples, helping them capture complex image features, optimize restoration strategies, and ultimately improve image recovery performance. For example, when the training dataset includes various types of images, such as landscapes, portraits, and dynamic scenes, CNN can not only learn the unique characteristics of different images but also adaptively adjust restoration strategies to improve the accuracy of detail recovery.

The introduction of deep learning has effectively addressed various distortions caused by image compression, particularly in standards such as VVC and HEVC, with notable improvements in handling quantization distortion, such as blocking artifacts and loop filtering distortions. Through feature learning, CNN can accurately restore the true visual appearance of images while prioritizing perceptual quality by focusing on human sensitivity to details, thereby enhancing the naturalness and realism of images. Compared with traditional filters, CNNs exhibit stronger distortion adaptability, allowing them to flexibly handle various distortions introduced by different encoding methods and compression rates. For example, a high compression rate may introduce significant blocking artifacts, while a lower compression rate may result in subtle noise or texture loss. CNN can intelligently distinguish between edge regions and smooth regions, enhancing details at edges while applying denoising in smooth areas to prevent artifacts caused by over-restoration [16,17,18,19,20]. Through continuous training, CNN can automatically adapt to various types of distortion, consistently optimizing image restoration and further enhancing visual quality.

CNN-based filters can be broadly categorized into in-loop filters and post-filters, each serving distinct roles in the image restoration process. Among these, CNN-based post-filtering [21,22,23,24] has demonstrated clear advantages over in-loop filtering [25,26,27,28,29] in several key aspects. It offers superior adaptability, interpretability, real-time performance, and generalization ability. For instance, post-filtering typically requires lower computational complexity and places fewer demands on hardware resources. It also shows strong adaptability to novel distortions, allowing for greater flexibility in addressing a wide range of image degradation issues. Furthermore, its improved interpretability enhances the potential for future development. In real-time scenarios, post-filtering enables more efficient processing with reduced latency, making it well-suited for time-sensitive applications. Lastly, it exhibits excellent generalization and reduced model dependency, achieving robust performance even in complex and diverse restoration tasks.

Building on the advantages of CNN-based post-filtering, this paper proposes a novel network called Dual-Path Residual Gating (DRGNet), which comprises two core components: an overfitting mitigation module and a gate-based denoising module. DRGNet is designed to fully leverage the characteristics of high-resolution images, effectively suppressing noise while preserving fine details. Our research focuses on two key aspects:

(I) Introducing a gating mechanism [30,31,32], allowing the module to dynamically allocate weights between noise and signal, effectively suppressing noise interference.
(II) The design of a residual connection module [33,34], reducing the risk of overfitting during the deep learning process and enhancing training stability.

In high-resolution video processing, challenges often arise due to the increased resolution. A higher pixel density means that noise has a more significant impact at the pixel level, and the distribution of noise is usually uneven. Traditional uniform denoising methods, such as mean filtering and Gaussian filtering, tend to cause detail loss and excessive smoothing, making the image blurry and reducing sharpness. Therefore, the Dual-Path Residual Gating (DPRG) block is adopted, which consists of two parallel branches. The main branch extracts the primary features of the input, while the gating branch calculates an adaptive mask using a sigmoid to control the weight of the main path, enabling more effective adaptive noise reduction. In detailed regions, the model effectively preserves the original information, preventing blurring caused by denoising; in high-noise regions, such as the sky and flat backgrounds, strong noise reduction is applied to remove excess noise. This achieves dynamic noise perception while effectively avoiding excessive smoothing. The addition of the LeakyReLU activation function compensates for potential negative feature loss caused by the gating mechanism, prevents neurons from losing gradients during training, improves convergence speed, and balances a noise suppression with texture preservation. As a result, the model suppresses noise while maintaining edge and texture details. Furthermore, the introduction of the residual mechanism ensures that the denoised image still retains the original video details, enhances training stability, and improves the model’s generalization ability, allowing it to maintain outstanding denoising performance across different scenarios.

This feature pyramid network is specifically designed to enhance feature representation across different levels, making it particularly suitable for high-resolution video denoising and feature extraction tasks. Its core objective is to fuse multi-scale information, enabling the model to simultaneously capture local details and global noise patterns, thereby improving denoising performance. Through 1 × 1 convolution, low-level features are aligned in terms of channels and then combined with high-level features to achieve cross-level feature fusion. Subsequently, a 3 × 3 convolution is applied to further enhance feature expression. This mechanism not only effectively integrates multi-scale information but also significantly improves edge sharpness and texture detail preservation, resulting in a denoised image that appears clearer and more natural.

The DRGNet model adopts the typical coding–decoding structure of convolutional neural networks, combined with noise suppression mechanism and multi-scale feature fusion. Overall, the model consists of three main parts: the front-end DPRG block sequence for feature extraction and noise suppression, the middle feature fusion module to simplify the feature pyramid for multi-scale feature fusion, and the tail module of the output phase to reconstruct the output. After the input goes through the initial convolution layer to extract the low-level features, multiple DPRG block stacks gradually denoise and refine the features. Then, the feature fusion module constructs feature pyramids from feature maps of different depths and integrate high-level semantics with low-level details to enhance the robustness of feature expression. Finally, the tail module processes the fused features and generates the final output of the model. The overall architecture reflects the design idea of “first noise suppression, then fusion, and multi-stage refining”, and the modules work closely together to maximize the removal of noise while maintaining image detail. The main research contributions of this paper are summarized as follows:

(I) A gating mechanism inspired by attention mechanisms is incorporated into the DPRG block to enhance feature selection. By multiplying the gating feature map with the main feature map, the model effectively retains essential features while suppressing noise. In parallel, the integration of residual networks mitigates the gradient vanishing problem, improving training stability and overall performance.
(II) As prior knowledge for image reconstruction, QP Maps play a crucial role in video coding. They guide the network in reconstructing image features and noise patterns, thereby enhancing reconstruction accuracy and overall network performance.
(III) The skip connection between the input image and the network’s residual output effectively preserves structural information and improves reconstruction accuracy through residual correction, leading to enhanced model performance and training efficiency.
(IV) Post-filtering in video encoding significantly improves image quality. The RA mode achieves efficient random access to video sequences by utilizing a structure that combines I, P, and B frames; the LDB mode focuses on minimizing latency; and the AI approach relies on I-frames to facilitate fast navigation. To balance the trade-offs among RA, LDB, and AI in terms of quality, compression efficiency, latency, and accessibility, the proposed neural network enhances video reconstruction fidelity across all three modes. The designed dual-path CNN post-processing framework is adaptable to various encoding scenarios and robust across a wide range of QP values.

The remainder of this paper is organized as follows. Section 2 explores the differences between CNN-based in-loop filtering and post-filtering, highlighting the advantages of the post-filtering approach adopted in this work. It also reviews the development of CNN-based video quality enhancement techniques and discusses the role of image denoising, including potential drawbacks associated with over-denoising. Section 3 presents a detailed description of the architecture and operational principles of the proposed neural network, DRGNet. Section 4 showcases the model’s effectiveness in improving image quality, supported by comprehensive performance evaluations and analysis. Finally, Section 5 outlines potential directions for future improvement and summarizes the key findings of this research.

## 2. Related Work

### 2.1. Development of the Neural Network Model

Neural networks have rapidly gained popularity across academia and industry due to their remarkable capabilities in solving complex tasks. Deep learning methods not only enhance flexibility and adaptability but have also made groundbreaking progress in fields such as natural language processing [35] and visual analysis and recognition [36], greatly advancing industry progress. Among these methods, CNN maintains an irreplaceable position due to its real-time processing capabilities, lightweight design, and efficiency in embedded applications. For example [37], in video and image quality enhancement tasks, neural networks have been cleverly applied to remove noise and artifacts, significantly improving the overall quality of images and videos. This deep learning-driven approach is becoming the mainstream choice for high-quality visual processing, accelerating technological innovation across various fields.

In the field of computer vision, image-denoising has become a classic and meaningful research topic. Traditional denoising methods have been widely applied in this area. Xu et al. [38] proposed a single-path CNN called DnCNN, which was the first to demonstrate the effectiveness of the residual learning strategy in image-denoising tasks. By training a residual model, DnCNN achieved more stable training performance during optimization and significantly improved denoising results. Building upon this, Zhang et al. [39] introduced FFDNet, which further refined the DnCNN architecture by proposing a fast and flexible denoising CNN capable of handling images with different noise levels. Meanwhile, Tai et al. [40] proposed MemNet, which employs a dense connection mechanism [41] within a deep learning framework. This design not only enhances the model’s stability during operation but also effectively captures short-term memory. Additionally, the model integrates a gating university, allowing it to adaptively learn the weights of different memory states, thereby further improving its denoising capability. Zhang et al. [42] proposed a CNN-based image denoising method that incorporates denoising priors into a model-driven optimization framework, effectively addressing various image restoration challenges. In real-world noise denoising tasks, Wu et al. [43] introduced RIDNet, which employs a residual network structure combined with a feature attention mechanism, further enhancing noise modeling capability and delivering outstanding denoising performance. However, relying solely on a single-path feature extraction approach presents inherent limitations in extracting image features. To overcome this, Tian et al. [44] proposed a dual-path CNN, which integrates batch renormalization to achieve complementary feature extraction, thereby improving denoising effectiveness. Furthermore, studies such as [45,46,47] have further explored the advantages of dual-path designs in denoising tasks. These methods leverage parallel feature extraction and fusion mechanisms, effectively enhancing the network’s ability to handle complex noise. Specifically, they utilize multi-scale convolution kernels, attention mechanisms, and feature fusion strategies to extract key information at different levels, significantly improving image denoising performance. However, these approaches commonly face challenges such as blurring or excessive sharpening of image edges and details. These issues are particularly pronounced in high-resolution image processing, where computational complexity significantly increases. High-resolution images require extensive pixel similarity computations, along with complex neighborhood operations and global calculations, further adding to the computational burden. Although recent advancements have made progress in improving the quality of images and videos, the improvement in high-resolution videos remains relatively slow compared with low-resolution videos. This highlights an urgent need for more efficient solutions. In Table 1, some of the recent works have been highlighted to provide a better comparison against our proposed method. Although most of the prior works focused on the HEVC codec, and an exact quantitative comparison is not possible with our works, we mainly studied their deep neural network architecture and, therefore, the core contribution of those studies.

### 2.2. Neural Network-Based In-Loop Filtering

In the field of video coding, numerous CNN-based methods have been proposed for in-loop filtering [25,26,27,28,29] to enhance image and video quality. Traditional approaches such as deblocking and SAO remain suitable for real-time applications due to their low computational complexity. However, these methods are mainly designed to address specific artifacts and often struggle to manage diverse types of noise, particularly in low bitrate or high-resolution scenarios where their performance becomes limited and the computational burden increases. With the rapid advancement of deep learning, neural networks have emerged as a promising solution in both post-processing [21,22,23,24] and in-loop filtering tasks. Leveraging powerful feature learning capabilities, these models effectively overcome the limitations of conventional techniques, offering significant improvements in visual quality and adaptability across varying encoding conditions.

Beyond the neural network technologies already embedded in loop filters, research has expanded to include post-filtering neural networks. Post-filtering neural networks leverage deep learning models to optimize decoded video, effectively reducing artifacts, enhancing details, and improving overall image quality. Compared with loop filtering neural networks, post-filtering neural networks can directly enhance the user viewing experience with more noticeable results. They also offer greater flexibility, independent of standards, and are not constrained by encoding efficiency. These unique characteristics make them particularly suitable for quality enhancement of high-resolution video content. In 4K and higher-resolution scenarios, CNN post-filtering can be applied after decoding, free from the limitations of in-video encoder filtering algorithms, thereby achieving more precise global optimization. It can remove compression artifacts, noise, and blurring based on the decoded video content, significantly improving video quality. In high-resolution video, these improvements in detail are crucial for the viewing experience. In contrast, loop filtering is limited by the encoding process, and its optimization effect is typically confined to error correction during the encoding stage. Post-filtering, however, can fully utilize the high-resolution video details after decoding, further enhancing visual quality.

### 2.3. Neural Network-Based Post-Filtering

The limitations of loop filtering highlight the advantages of CNN post-filtering in removing artifacts and noise. During the compression and decoding of high-resolution videos, common artifacts such as block effects, mosaic effects, and noise issues are especially prominent, and noise handling is particularly crucial. CNN post-filtering, through deep neural networks, can automatically adapt to the diverse characteristics of image noise, effectively removing noise while preserving image clarity and details, especially performing excellently in low-light or complex scenes. Compared with loop filtering, post-filtering is more refined in removing complex compression artifacts and noise, particularly in high-resolution videos where traditional loop filtering may fail to fully remove noise or restore details due to computational resource limitations or algorithm simplicity. Therefore, CNN post-filtering’s advantages are more pronounced in high-resolution video processing.

**Figure 1:**
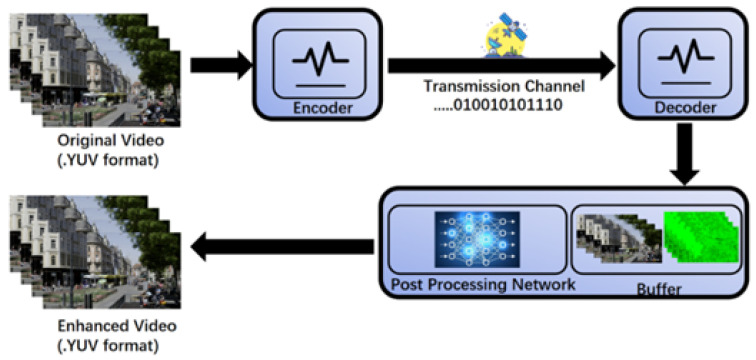
Deep learning-based post-processing approach in typical coding workflow.

Figure 1 illustrates the complete workflow of video encoding post-processing. In this workflow, a refinement step based on CNN is introduced after video decoding to improve the quality of the reconstructed video. This technique uses artificial intelligence to optimize the decoded video, analyzing and refining the reconstructed content through deep learning models, thereby significantly enhancing the visual quality of the video. Specifically, the main steps of the experiment are as follows: the input is an uncompressed raw video in YUV format, which is sent to the encoder for compression. The encoder compresses the original video, significantly reducing its data size to make it suitable for transmission. Then, the compressed video data is transmitted in binary form, such as 01010101110, through the transmission channel. During transmission, the data may be affected by noise or signal loss, potentially causing errors. The decoder restores the received binary data into a video stream. However, due to potential losses during transmission, the quality of the decoded video may degrade.

To address this issue, the experiment introduces a post-processing network as a key component. The decoded video is passed through this deep learning-based network to enhance and restore visual quality [56,57,58,59]. This step effectively reduces distortions caused by compression and transmission. The optimized video is then stored in a buffer for playback or further use. The final output is an enhanced YUV-format video, which shows superior visual quality compared with the original decoded video. This process highlights the potential and value of AI-driven post-processing technologies in video reconstruction, demonstrating their ability to significantly improve video quality after decoding. This process highlights the potential and value of AI-driven post-processing technologies in video reconstruction, demonstrating their ability to significantly improve video quality after decoding. In particular, the enhancement of high-resolution content has become a critical focus of AI-driven post-processing, as traditional methods face limitations in flexibility and computational efficiency when applied to high-resolution video enhancement.

## 3. Proposed Method

### 3.1. Development of the Neural Network Model

In our prior work, we proposed the Post-Processing Feature Fusion (PPFF) model to address post-processing challenges in video coding. In the PPFF model proposed by [52], the preprocessed video data is fed into the core module for further optimization. The core module consists of a neural network that utilizes deep learning to extract features from the provided video data, achieving a significant improvement in video quality and meeting the optimization objectives of the experiment. The first step of the network structure involves using a 1 × 1 convolution layer in the head module to map the input image features to a fixed 128 channels. This process is used to adjust the number of channels and also lays a solid foundation for subsequent deep feature extraction. It ensures that the model can efficiently handle the input data and provide more precise optimization results. The features extracted from the head module are then passed to the backbone module for further processing. The backbone module consists of several feature extraction modules, each based on a 3 × 3 convolution filter structure with 128 output channels.

The highlight of the original PPFF model lies in using simple 3 × 3 convolution layers to extract image features, with residual features being added layer by layer from each convolution. This approach maximizes the capture of image information. However, this feature stacking method, while improving the average quality of all videos in the test set, has a limited impact on improving the quality of high-resolution videos. Only a certain degree of quality improvement is achieved in low-resolution videos through feature aggregation. The improved model proposed in this paper significantly enhances the feature extraction capacity of each convolution kernel by introducing residual networks and gating mechanisms. The residual structure deepens the network while effectively mitigating the vanishing gradient problem. The gating mechanism, through dynamic weight adjustments, effectively removes noise in high-resolution videos. These advancements significantly improve the quality of high-resolution videos, overcoming the limitations of traditional approaches that struggle with slow progress in quality enhancement at higher resolutions.

### 3.2. Proposed Dual-Path Residual Gating

#### 3.2.1. Architecture Overview of DRGNet

**Figure 2:**
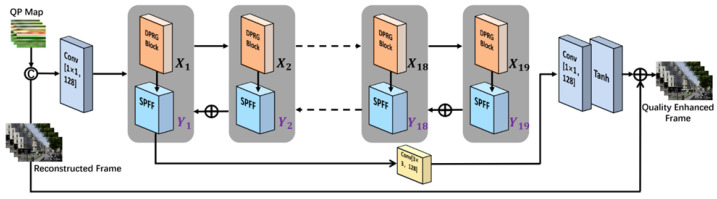
DRGNet model of the main composition diagram.

Figure 2 shows the overall architecture of the proposed DRGNet. The design of DRGNet showcases strong performance in image denoising and enhancement tasks across various scenarios. To address the specific challenges posed by images of different resolutions, the model is designed for high-resolution image denoising, effectively removing noise while preserving visual details. The final experimental results closely align with our initial expectations, thoroughly validating both the effectiveness and reliability of the proposed architecture. First, the input is a tensor with the shape of 240 × 240, containing image data with six channels. The first layer of the model is a convolutional layer that maps the input image channels to 128 channels, laying the foundation for subsequent feature extraction.

The middle part of DRGNet consists of multiple DPRG blocks, each containing two parallel convolutional layers and a gating mechanism, where each path processes features through a 3 × 3 convolutional layer. The main feature branch focuses on extracting critical information and capturing important features from the data. The gating mechanism, using the sigmoid activation function, adaptively selects which features should be retained, thus effectively enhancing feature quality while suppressing noise interference. Each gating block is combined with residual connections to ensure smoother information flow throughout the network. In DRGNet, the gating mechanism and attention mechanism share a very similar design logic, particularly in terms of feature selection and information weight distribution. Although the implementation methods differ, their core idea is the same: helping the network focus on important features while suppressing irrelevant or redundant information. Furthermore, the inclusion of the residual model significantly enhances the stability and robustness of the network.

Additionally, DRGNet strengthens the fusion of multi-scale features through a Special Pyramid Feature Fusion block (SPFF). The SPFF module propagates high-level features downward in a top-down manner and performs layer-wise feature fusion, effectively preserving deep semantic information while enhancing spatial details in lower-level features. In addition, the module is specifically optimized based on the operational logic of DRGNet to further improve the effectiveness of feature fusion. By fusing feature layers at different scales, the model can capture multi-level information from details to the global context, further improving the restoration of image details and denoising capability.

The tail of the network consists of a convolutional layer and a Tanh activation function, used to generate the final image output. Additionally, the tail includes a dropout layer to effectively prevent overfitting. Finally, the model supports residual connections, adding the input image to the output processed by the tail. This design helps the network better learn the differences between the input and output, avoiding the loss of the original image information.

Overall, the DRGNet model demonstrates excellent performance in the video frame denoising enhancement task through noise gating and residual learning mechanisms combined with a specialized SPFF. Its design, through multi-level feature extraction and fusion, reduces noise and restores image details. Experimental results confirm that the model design achieves the anticipated results, making it particularly suitable for denoising and enhancement applications in high-resolution images.

#### 3.2.2. DRGNet Component

**Figure 3:**
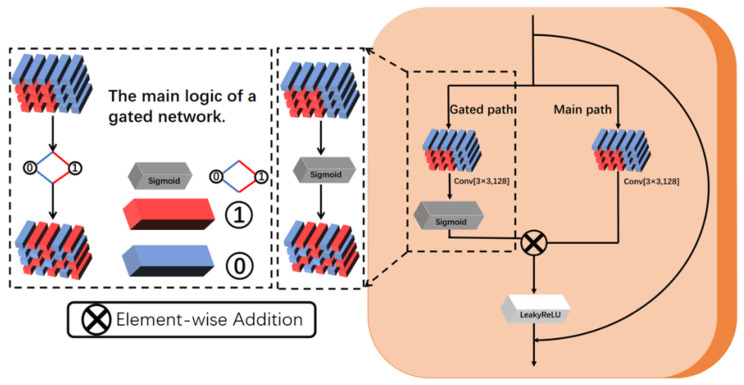
Block diagram of DPRG.

As shown in Figure 3, the internal structure adopts a parallel design, with two 3 × 3 convolutional layers arranged in parallel, forming the main feature extraction branch and the gating branch. The gating branch combines convolutional operations and an S-shaped activation function to generate gating weights. The output from the main feature branch is then multiplied by the gating weights, dynamically adjusting the importance of the features. Subsequently, the residual connection adds the input to the processed output and applies the LeakyReLU activation function for further processing, enhancing the non-linear representation ability. This process is repeated layer by layer in each feature extraction module until deep feature extraction is completed, ensuring stable convergence during deep training and making the extracted feature information more refined and rich. Through rigorous experimental analysis, we determined that using 19 feature blocks is the optimal configuration for the model. This number has been carefully adjusted to optimize the model’s effectiveness across different experimental scenarios while maintaining flexibility to adapt to specific experimental requirements. Our model follows the design principles of residual networks, maintaining simplicity while effectively mitigating overfitting issues in deep learning. The residual network structure improves gradient flow, significantly enhancing the stability and learning ability of the model, thus boosting the overall performance of deep optimization. The tail module processes the output channels from the main module.

As shown in Equation (1) and Figure 3, the convolution operation of the main path and how it is computed on the feature map. *Conv*_*main*_(x) means that the main convolution operation is performed on the input feature graph *X* to generate the output feature graph *main_features*. The equation is given as follows:

(1) $${\mathrm{Conv}}_{\mathrm{main}}\left(x\right)=W_{\mathrm{main}}*X.$$

Equation (2) is a more detailed derivation of Equation (1) and is expressed as

(2) $${\mathrm{main}}_{\mathrm{features}}\left[c,i,j\right]=\sum_{m=-k}^{k}\sum_{n=-k}^{k}W_{\mathrm{main}}\left[c,m,n\right]\cdot X\left[c,i+m,j+n\right].$$

The operation sums the input feature map *X* with local weights, where $W_{\mathrm{main}}\left[c,i+m,j+n\right]$ denotes the value of the input feature map at channel *c* and coordinate (i+m,j+n). The term $W_{\mathrm{main}}\left[c,m,n\right]$ represents the weight of the main convolution kernel at channel *c* with a relative offset of (m,n). Double summation $\sum_{m=-k}^{k}\sum_{n=-k}^{k}$ traverses the entire convolution kernel window of (2K+1)×(2K+1), summing the product of the weights and the input eigenvalues at each position. *K* stands for the radius of the convolution kernel, which means the size of the convolution kernel is (2K+1)×(2K+1). For example, when the convolution kernel is 3×3, the immediate summation range is −1 to 1, where m=−K to *K* represents the traversal of the convolution kernel in the vertical direction (row direction), and n=−K to *K* indicates the traversal range in the horizontal direction (column direction). Finally, it is calculated that ${\mathrm{main}}_{\mathrm{features}}\left[c,i,j\right]$ is the value of position *X* at channel *c* after the convolution of the input feature graph (i,j). The gated branch generates gating weights to dynamically adjust the contribution of the main features. Its basic convolution layer is a standard 3×3 convolution layer. According to Equations (3) and (4), this operation is essentially consistent with the main path and is represented as

(3) $${\mathrm{gating}}_{\mathrm{features}}={\mathrm{Conv}}_{\mathrm{gate}}\left(x\right),$$

(4) $${\mathrm{gating}}_{\mathrm{features}}\left[c,i,j\right]=\sum_{m=-k}^{k}\sum_{n=-k}^{k}W_{\mathrm{gate}}\left[c,m,n\right]\cdot X\left[c,i+m,j+n\right].$$

According to Equation (5), the output of the convolution layer in the gated branch is processed using the sigmoid activation function, which maps feature values to the range [0, 1], and is expressed as

(5) $${\mathrm{gating}}_{\mathrm{feature}}=\sigma_{\mathrm{sigmoid}}\left({\mathrm{Conv}}_{\mathrm{gate}}\left(x\right)\right).$$

These weights represent the importance of each feature. Utilizing the sigmoid function, each pixel value in gating features can be interpreted as an importance weight for that position, where values close to 1 denote higher importance in gating features [c, i, j] → 1, while values close to 0 represent suppressed features in gating features [c, i, j] → 0. Equation (6) is as follows:

(6) gated_output(c,i,j)=main_features(c,i,j)·gating_features(c,i,j).

Then, the output of the main feature branch is dynamically fused with the gating weights through element-wise multiplication, amplifying important features while suppressing irrelevant or noise-affected features.

The fused features are further processed by the LeakyReLU activation function, which not only introduces non-linear representation capability but also allows small negative values to pass through, effectively avoiding the “dead neuron” problem commonly encountered in traditional ReLU. This mechanism enhances the network’s ability to learn effective features in noisy environments. The symbol Z represents the combined operation of residual connection and non-linear activation in the BPRG module, as shown in Equation (7):

(7) $$Z=\mathrm{LeakyReLU}({\mathrm{gated}}_{\mathrm{output}}+x).$$

According to the residual connection mechanism, the input features are directly added to the processed output, effectively preserving the original information while reducing noise interference. This approach also improves the stability and representational capacity of the network. The final output is given by Equation (8):

(8) $$\mathrm{output}=\sigma_{\mathrm{LeakyReLU}}\left(\mathrm{Conv}\left(x,W_{\mathrm{main}}\right)\cdot \sigma_{\mathrm{sigmoid}}\left(\mathrm{Conv}(x,W_{\mathrm{gate}})\right)+x\right).$$

This module effectively suppresses noise, dynamically optimizes feature importance, and significantly enhances the network’s non-linear representation capability and deep feature extraction performance through the combined effect of main path feature extraction, dynamic gating adjustment, and residual connections.

**Figure 4:**
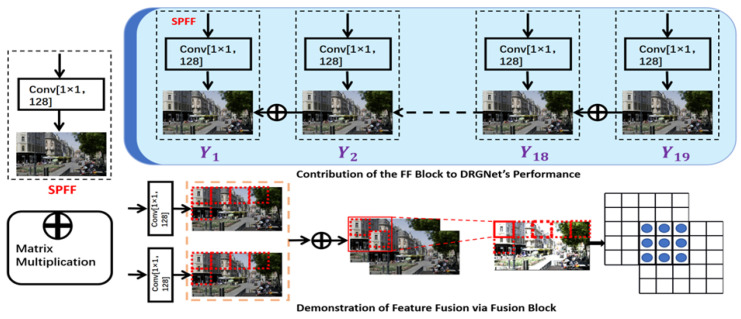
Block diagram of SPFF.

As shown in Figure 2 and Figure 4, an SPFF (Special Pyramid Feature Fusion) module is appended after each DPRG block to further extract image features captured by the DPRG blocks. This design effectively reduces the loss of image features during model training. By leveraging pixel-level addition, it emphasizes important details within the image, thereby enhancing the completeness and accuracy of feature extraction. The SPFF structure integrates a 1×1 convolutional filter and three output channels and employs the Tanh activation function to constrain the output values within the range of [−1,1]. Finally, the tail module further refines the decoded image using a residual connection mechanism. The image reconstructed by the head module is directly passed to the output of the tail module via a skip connection. This residual update mechanism corrects potential artifacts introduced during feature transformation and reconstruction, ensuring that the main information in the decoded image remains intact. This forms the core objective of the method proposed in this study.

Equation (9) defines the computational process of the SPFF. The entire process can be represented by the following equation:

(9) $$\begin{array}{cc} P_{i} & ={\mathrm{Conv}}_{3\times 3}\left({\mathrm{Conv}}_{1\times 1}\left(X_{i}\right)+P_{i+1}\right),\\ i & =n-1,n-2,\ldots ,1. \end{array}$$

Here, $P_{i}$ denotes the final output feature map at each layer, $X_{i}$ represents the input feature map from the backbone network, ${\mathrm{Conv}}_{1\times 1}$ ensures channel dimensional consistency to enable seamless multi-level feature fusion, and $P_{i+1}$ indicates the fused feature from the previous layer. Let the output feature of the *i*th layer be $P_{i}$, with its corresponding original input feature denoted as $X_{i}$.

The DPRG block incorporates residual connections, a key principle derived from ResNet, which are essential for preserving information and stabilizing the overall network structure. Their introduction not only ensures the stability of training during the deep learning process but also effectively alleviates issues such as vanishing gradients and feature degradation common in deep networks. By incorporating residual connections, the network minimizes the risk of errors or excessive suppression of important features during the primary feature processing path. Furthermore, the residual structure plays a crucial role in feature fusion, avoiding issues such as edge blurring and over-sharpening commonly encountered in traditional denoising methods while preserving high-frequency details and essential feature information. The combination of the gating and main paths in the DPRG block enables effective feature extraction and dynamic adjustment, allowing the network to accurately locate and suppress noise areas.

During model operation, residual connections supplement the original information that may not have been fully processed by the gating and main paths, ensuring the integrity of the information. When the output of the gating and main paths attenuates in low-weight areas, the residual connection effectively suppresses noise. In high-weight areas, it enhances key features, ensuring the effectiveness and robustness of the output. This collaborative effect makes the DPRG block excel in both noise suppression and feature enhancement.

The model integrates the SPFF structure with the DPRG block, significantly enhancing the overall feature extraction capability while maximally preserving detailed information in the feature maps during the training process. By introducing the gating system, the model has achieved outstanding results in high-resolution video, fully exploiting the inherent characteristics of high-resolution video and greatly improving processing effectiveness.

The logic of the gating system is derived from the core concept of attention mechanisms, dynamically assigning weights based on the learned importance of different features to highlight key regions. Unlike the PPFF model, DRGNet replaces the original simple backbone with a more complex structure while also improving the feature pyramid model and integrating it into DRGNet to establish a more efficient feature fusion mechanism.learning process and minimizes feature map loss during training. The dynamic weight allocation method has been widely applied in image noise optimization tasks. The gating system assigns higher weights to critical regions in the image, such as edges, textures, and structural components, thereby preserving and further enhancing these essential features. As a result, DRGNet significantly improves image quality by effectively suppressing noise and enhancing fundamental visual details.

#### 3.2.3. Training Strategy

The BVI-DVC [60] dataset contains 800 videos, covering resolutions from 270 p to 2160 p, and is used for neural network model training. Video compression is based on the JVET neural network-based video coding (NNVC) Common Test Conditions (CTC) standard and is processed using RA, LDB, and AI configurations. The BVI-DVC dataset was not divided into test sets or multiple independent data partitions; all evaluations were conducted using separate JVET NNVC CTC test sequences. The original video format is MP4, which is then converted to YUV format, adopting 4:2:0 chroma subsampling and 10-bit depth. In data preprocessing, to ensure a reasonable distribution of multi-resolution videos, 10 frames are extracted from each video, accumulating 8000 frames for training. However, due to inconsistent frame sizes across videos of different resolutions, the proposed network cannot directly process these heterogeneous inputs. Therefore, chroma channels are upsampled by a factor of 2 to match the spatial resolution of the luma channel. Subsequently, a randomly cropped patch of size 240×240 is extracted from each video as input to the model. According to the JVET NNVC CTC guidelines, the experiments are configured under three settings: RA, LDB, and AI. Under each configuration, five independent CNN models are trained, each corresponding to a specific quantization parameter (QP). Based on the strategy shown in Table 2, the selected QP values are 22, 27, 32, 37, and 42. Regarding the loss function, this study adopts the Mean Squared Error (MSE) as the primary optimization objective. MSE is simple and computationally efficient, making it well-suited for enhancing compressed images, particularly for structure-aligned reconstruction tasks. During training, supervision is applied using QP-normalized inputs, which enhances the model’s stability and generalization under various compression intensities. The MSE loss does not require additional hyperparameter tuning. Its theoretical soundness and practical effectiveness have been validated in this study, especially in terms of structural detail preservation and overall image fidelity. Furthermore, it provides a solid foundation for integrating advanced losses, such as perceptual and edge-aware loss, in future work. All models are optimized using the Adam optimizer with a learning rate of $10^{-4}$ and trained for 200 epochs. During optimization, the hyperparameters are set to $\beta_{1}=0.9$ and $\beta_{2}=0.999$ to compute the moving average of gradients, ensuring stable convergence.

**Figure 5:**
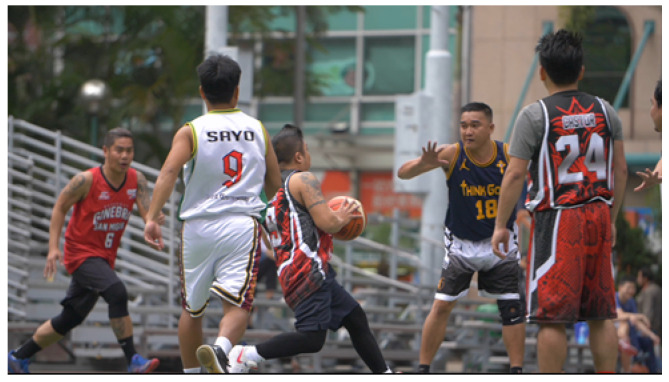
Raw high-resolution frame 3840 × 2176 from the BVI-DVC training set, extracted from the video ABasketballGoalScoredS2Videvo and used during the training phase of the proposed model.

As shown in Figure 5, a raw high-resolution frame with a resolution of 3840 × 2176 is extracted from the video ABasketballGoalScoredS2Videvo in the BVI-DVC training set. This frame is used during the training phase of the proposed model to preserve the original visual fidelity for supervised learning.

Table 3 and Table 4 enlist the brief descriptions of the training and testing datasets, respectively, used in this study. Table 3 shows the distribution and encoding characteristics of the BVI-DVC training dataset. The dataset includes a total of 800 videos, evenly divided across four different resolutions: 3840 × 2160, 1920 × 1080, 960 × 544, and 480 × 272. Each resolution group contains 200 videos, and 2000 frames are extracted for training. All videos are encoded with a bit depth of 10 and use 4:2:0 chroma subsampling. This balanced resolution distribution ensures that the training data covers a wide range of spatial complexities, helping the model to learn robust features across various video qualities and resolutions. Table 4 summarizes the video classification and encoding parameters of the JVET NNVC CTC test set. The dataset includes videos from four classes: A1, A2, B, and C, which differ primarily in spatial resolution. Classes A1 and A2 both have a resolution of 3840 × 2160 but contain different sequences, with three videos each and frame counts of 1314 and 1400, respectively. Class B consists of five videos at 1920 × 1080 resolution, totaling 2802 frames, while Class C includes four videos at 832 × 480 resolution with 1903 frames in total. All sequences are encoded using 10-bit depth and 4:2:0 chroma subsampling, consistent with the JVET CTC configuration. This test set provides a diverse set of resolutions and content types for evaluating the performance of neural post-processing models under standardized video coding conditions.

## 4. Results

### 4.1. Experimental Environment

The testing and evaluation of the proposed method in this study are based on the standard test sequences defined in the JVET NNVC CTC guidelines. These sequences were not used during the training phase, ensuring a complete separation between training and testing data, thereby guaranteeing the fairness and generalizability of the experimental results. According to the JVET NNVC CTC guidelines, these sequences are divided into six categories: A1, A2, B, C, D, and E were used to evaluate the RA, LDB, and AI configurations. The QP for all test configurations was set to 22, 27, 32, 37, and 42 to comprehensively assess the performance of the method under varying compression levels. RA configurations are detected using A1, A2, B, and C class sequences. This mode is designed for scenarios requiring quick random jumps or access, such as video on demand, live streaming, and broadcast television. LDB configuration uses sequences from classes B and C for testing. This mode aims to enhance compression efficiency while minimizing latency, making it suitable for real-time communication and live-streaming scenarios. The AI configuration test used sequences from categories A1, A2, B, and C. All frames are independently encoded as I-frames, highlighting the independence of the frame. This mode is ideal for editing, archiving, and scenarios requiring high flexibility, as no inter-frame references are needed for decoding. These configurations ensured a comprehensive evaluation of the method performance in various scenarios, compression intensities, and sequence categories. The experiment uses PyTorch [61] as a deep-learning framework running on an Ubuntu-based system. The system is equipped with powerful hardware, including four AMD Ryzen Threadripper PRO 3955WX 16-Core processors from Advanced Micro Devices, Inc., Santa Clara, CA, USA, and four high-performance NVIDIA GeForce RTX 3090 GPUs from NVIDIA Corporation, Santa Clara, CA, USA.

**Figure 6:**
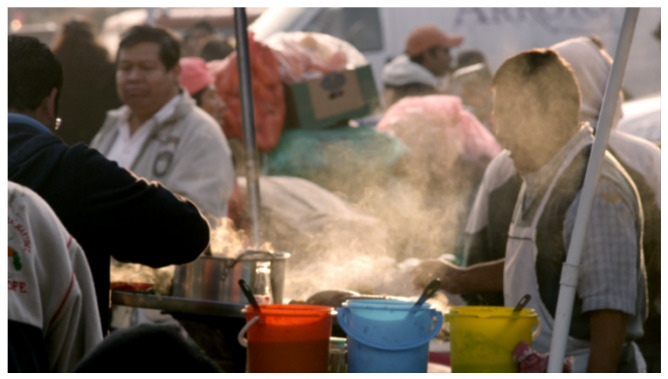
Example frame from a high-resolution testing video in the JVET NNVC CTC dataset.

As shown in Figure 6, an uncompressed high-resolution frame with a resolution of 3840 × 2160 is extracted from the video FoodMarket4 in the JVET NNVC CTC test set. This frame is used during the evaluation phase to assess the performance of the proposed model on high-resolution testing data.

### 4.2. Experimental Evaluation Method

The combined use of PSNR and Bjøntegaard Delta Rate (BD-Rate) provides a comprehensive evaluation of video coding performance, effectively reflecting the balance between visual quality and compression efficiency. In this study, the output quality of the proposed method was compared with that of the decoded frames generated by the codec software. PSNR was used to quantify the differences between the original and reconstructed frames, offering a direct assessment of visual quality, while BD-Rate was used to measure the relative compression efficiency under equivalent quality levels.

PSNR is used as the evaluation metric, and its calculation is defined as shown in Equation (10), expressed as

(10) $$\mathrm{PSNR}=10\cdot \log_{10}\left(\frac{L^{2}}{\frac{1}{\left|\Omega \right|}\sum_{(x,y)\in \Omega }{\left[I\left(x,y\right)-\hat{I}\left(x,y\right)\right]}^{2}}\right)$$

The output quality of the proposed method was compared with the output quality of decoded frames generated by the VVenC software version 1.10.0 PSNR quantifies differences between original and reconstructed frames, providing an assessment of reconstructed video quality. A higher PSNR value indicates reconstructed frames are closer to original frames, reflecting better compression performance and higher quality.

In this case, MSE (Mean Squared Error) in Equation (11) is used to calculate the average squared error across all pixel positions between the original image and the reconstructed image.

(11) $$\mathrm{MSE}=\frac{1}{\left|\Omega \right|}\sum_{(x,y)\in \Omega }{\left[I\left(x,y\right)-\hat{I}\left(x,y\right)\right]}^{2}$$

Here, α represents the set of all pixel positions in the image, and |α| denotes the total number of pixels. I(x,y) is the pixel value of the original image at position (x,y), while $\hat{I}\left(x,y\right)$ represents the corresponding pixel value in the reconstructed image. The term $I\left(x,y\right)-\hat{I}\left(x,y\right)$ captures the pixel-wise difference between the original and reconstructed images, and ${\left[I\left(x,y\right)-\hat{I}\left(x,y\right)\right]}^{2}$ is the squared error at that location, which penalizes larger deviations more severely. By summing the squared errors across all pixel positions and dividing by the total number of pixels, the Mean Squared Error (MSE) quantifies the overall average reconstruction error between the original and compressed images.

BD-Rate is a key metric in the field of video encoding, used to evaluate compression efficiency by comparing the bitrate of a video codec with that of a reference codec or technology. It calculates the average percentage difference in bitrate across different quality levels or quantization parameters. A lower BD Rate value indicates better compression performance.

According to Equation (12), BD-Rate is expressed as a percentage, providing a concise summary of encoding efficiency, making it an important tool for evaluating and comparing the performance of different codecs and techniques in video encoding.

(12) $$\mathrm{BD}\text{-}\mathrm{Rate}=\left(10^{\frac{1}{P_{\max}-P_{\min}}\int_{P_{\min}}^{P_{\max}}\left(\log_{10}R_{B}\left(P\right)-\log_{10}R_{A}\left(P\right)\right)dp}-1\right)\times 100\%.$$

Because of its intuitiveness and practicality, BD-Rate has been widely adopted in the video coding field.

According to Equation (13), the expression is a cubic polynomial fitting function used to model the relationship between the bitrate R and the quality metric P, typically PSNR or SSIM.

(13) $$\log_{10}R=aP^{3}+bP^{2}+cP+d.$$

By fitting the discrete data points, a continuous mathematical model can be established, facilitating subsequent integration calculations.

According to Equation (14), $R_{A}\left(P\right)$ and $R_{B}\left(P\right)$ represent the bitrates of encoding schemes A and B, respectively, at a given quality metric *P*.

(14) $$\Delta =\frac{1}{P_{\max}-P_{\min}}\int_{P_{\min}}^{P_{\max}}\left(\log_{10}R_{B}\left(P\right)-\log_{10}R_{A}\left(P\right)\right)dP$$

This equation calculates the average difference in logarithmic bitrate between the two fitted functions over a specified quality range $\left[P_{\min},P_{\max}\right]$.

Equation (15) defines the final expression of the BD-Rate, which indicates that

(15) $$\mathrm{BD}\text{-}\mathrm{Rate}=\left(10^{\Delta }-1\right)\times 100\%.$$

The average difference Δ in the logarithmic space is converted into the actual bitrate difference percentage, which is referred to as the BD-Rate.

### 4.3. Experimental Environment

Table 5, Table 6 and Table 7 provide a detailed breakdown of the compression performance of the proposed architecture, highlighting its superior efficiency under RA, LDB, and AI configurations. The compression performance is evaluated using the BD-Rate metric recommended by JVET, where lower values indicate higher encoding efficiency. According to the experimental results of the RA configuration in Table 5, the BD-Rate values for the component of the luma (i.e., Y component) and the component of the chroma (i.e., U and V components) were calculated separately. The A1 and A2 groups comprise high-resolution video sequences, each with a resolution of 3840 × 2160. In contrast, the B and C groups consist of low-resolution sequences, with resolutions of 1920 × 1080 and 832 × 480, respectively. The average BD-Rate for the A1, A2, and B and C video groups were 5.87%, 16.07%, and 16.41%, respectively. The test video group includes a variety of videos ranging from high resolution to low resolution. Notably, in high-resolution video scenarios, the proposed neural network model demonstrated significant advantages, with the quality improvement rate far exceeding that of low-resolution videos. For example, in the A2 sequence, the BD-Rate of the Y component in the DaylightRoad video decreased by 8.75%, while in the CatRobot video, the BD-Rate of the Y component decreased by 7.61%. Among them, the DaylightRoad video achieved the best reduction in BD-Rate for the Y component in the entire RA video group, further verifying the effectiveness and significance of the proposed model in high-resolution video encoding scenarios.

According to the experimental results of the RA configuration in Table 5, the BD-Rate values for the component of the Y and the component of the U and V were calculated separately. The average BD-Rate for the A1, A2, and B and C video groups were 5.87%, 16.07%, and 16.41%, respectively. The test video group includes a variety of videos ranging from high-resolution to low-resolution. Notably, in high resolution video scenarios, the proposed neural network model demonstrated significant advantages, with the quality improvement rate far exceeding that of low-resolution videos. For example, in the high-resolution sequence, the BD-Rate of the Y component in the DaylightRoad video decreased by 8.75%, while in the CatRobot video, the BD-Rate of the Y component decreased by 7.61%. Among them, the DaylightRoad video achieved the best reduction in BD-Rate for the Y component in the entire A video group, further verifying the effectiveness and significance of the proposed model in high-resolution video encoding scenarios.

Table 6 presents the coding performance of the proposed framework under the LDB configuration. The BD-Rate reductions for the Y component and U and V components were calculated, with average reductions of 6.84%, 27.86%, and 27.69%, respectively. In specific video groups, the BD-Rate for the Y component of group B videos decreased by 5.84%, while for group C videos, it decreased by 7.53%. These results indicate that the proposed framework exhibits outstanding improvements in high-resolution videos, where other neural networks often struggle to achieve significant optimization. This experimental result not only aligns with the design objectives of the model but also validates its potential to achieve efficient compression and significant quality enhancement in practical applications. The results are highly consistent with the initial design motivations and performance expectations.

Table 7 presents the coding performance of the proposed framework under the AI configuration. The average BD-Rate reduction for the Y component and U and V components reaches 5.80%, 10.10%, and 12.41%, respectively. In terms of performance across different video groups, the 3840 × 2160 video group A1 achieves an average BD-Rate reduction of 5.04%, while group A2 achieves an even higher reduction of 6.66%, both significantly outperforming lower-resolution video groups. Notably, within group A2, the BD-Rate reduction for the Y component of the CatRobot and DaylightRoad videos reaches as high as 8.59% and 9.11%, respectively, further highlighting the outstanding performance of DRGNet in high-resolution video processing. At the same time, the model demonstrates strong adaptability and generalization across different environments, further proving its efficiency and comprehensiveness in video coding optimization.

As shown in Table 8, we present a comprehensive evaluation of the proposed model in terms of PSNR metrics, providing a more holistic assessment of its performance. The PSNR results cover three representative VVC coding configurations: RA, LDB, and AI. For each configuration, we report results across four standard test classes: A1, A2, B, and C. Each test class includes PSNR improvement values under five QP settings: 22, 27, 32, 37, and 42, enabling fine-grained analysis of the model’s behavior under varying compression strengths. The experimental results demonstrate that the model achieves consistent and stable PSNR gains across all VVC configurations. Moreover, the enhancement in chrominance components U and V is also notable, indicating that the proposed method not only improves the luma Y quality but also effectively suppresses color-related artifacts. Overall, the PSNR improvements are substantial, and subjective visual results further validate the model’s ability to restore fine details in compressed frames.

### 4.4. Perceptual Quality Evaluation Results

**Figure 7:**
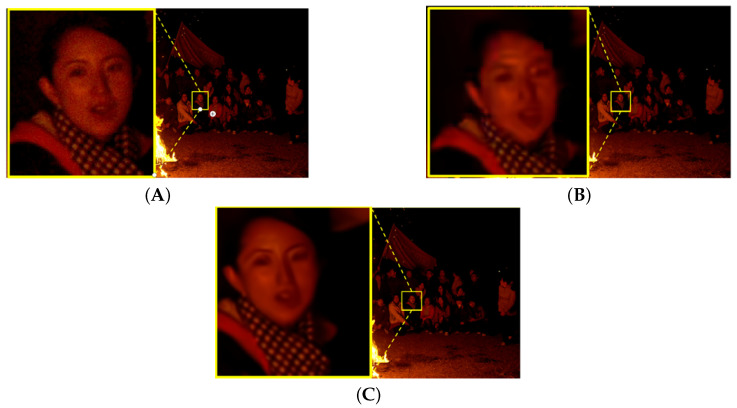
Sequence of Campfire with 3840 × 2160 (**A**) original, (**B**) VVC compressed, and (**C**) proposed approach for AI configuration with QP 32.

**Figure 8:**
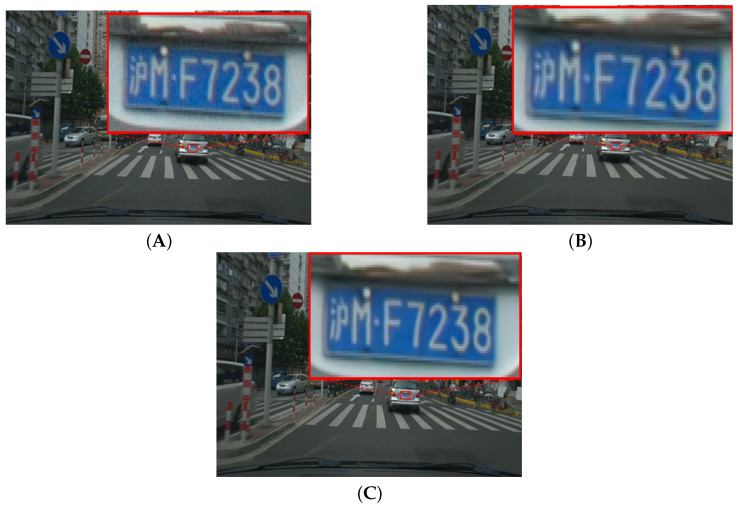
Sequence of Daylightroad with 3840 × 2160 (**A**) original, (**B**) VVC compressed, and (**C**) proposed approach for AI configuration with QP 32.

**Figure 9:**
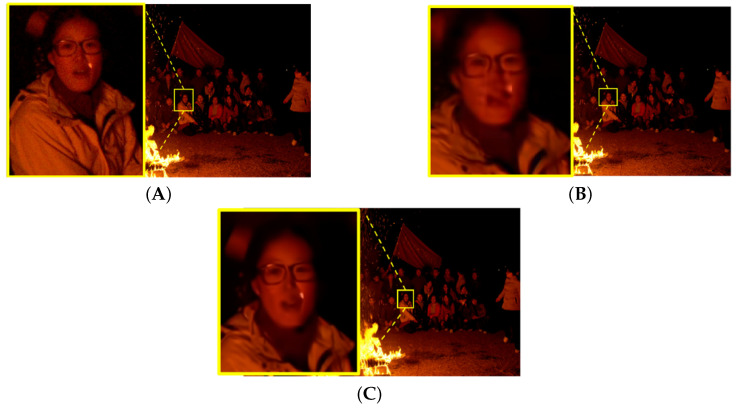
Sequence of Campfire with 3840 × 2160 (**A**) original, (**B**) VVC compressed, and (**C**) proposed approach for RA configuration with QP 32.

**Figure 10:**
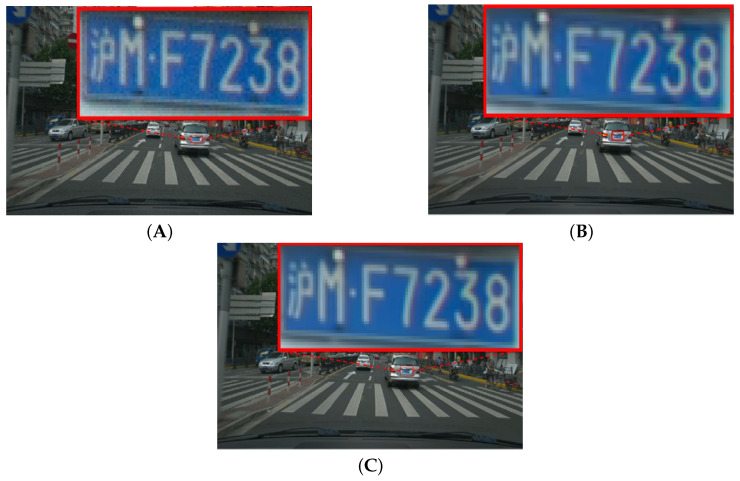
Sequence of Daylightroad with 3840 × 2160 (**A**) original, (**B**) VVC compressed, and (**C**) proposed approach for RA configuration with QP 32.

**Figure 11:**
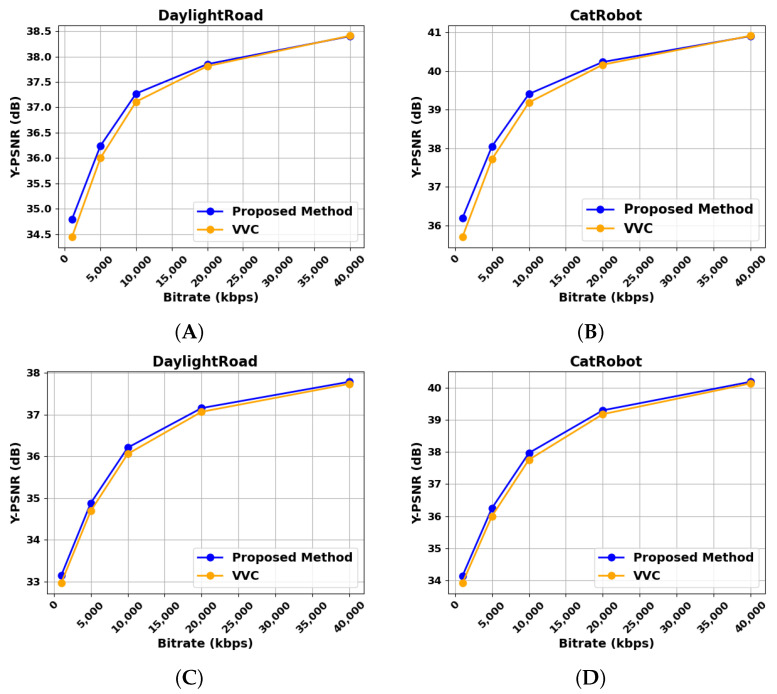
(**A**,**B**) are the comparison of high-resolution video in the AI configuration with video in the VVC structure. (**C**,**D**) are the comparison of high-resolution video in the RA configuration with video in the VVC structure.

Figure 7, Figure 8, Figure 9 and Figure 10 illustrate the visual quality comparison under the AI and RA configurations. In these images, we compare the original video frames and VVC-compressed frames, and the results are enhanced by the deep learning-based neural network. The evaluation is conducted at a QP value of 32, ensuring an objective assessment of visual quality improvements. Figure 7 presents the Campfire sequence under the AI configuration, where a noticeable reduction in noise around text edges and refinement of smooth edges can be observed. Our method achieves a PSNR gain of 0.39 dB over VVC, leading to improved visual perception. Figure 8 showcases the DaylightRoad sequence under the AI configuration, where a significant reduction in noise around numerical regions is evident, enhancing overall detail representation. The PSNR gain is 0.8 dB. Similarly, the proposed method reduces noise around numerical elements while enhancing the brightness of the blue background, thereby revealing more intricate details. In summary, under the QP 32 compression setting, our method demonstrates superior suppression of compression artifacts in both RA and AI configurations while effectively balancing high and low compression environments. This approach successfully reduces the bitrate while significantly improving visual quality, further validating the effectiveness of neural networks in video enhancement tasks and showcasing their great potential in optimizing compressed video quality. DRGNet model and VCC, in terms of Y performance in high-resolution videos within the RA environment, clearly demonstrate the significant improvement achieved by DRGNet. Notably, as the bitrate decreases, the noise in the video gradually increases, making changes in visual quality more pronounced. Figure 11C,D further indicate that in the RA configuration, the DRGNet model continues to deliver remarkable performance in high-resolution video processing. In the low bitrate range from 5000 to 20,000 kbps, the DRGNet model achieves significantly better gains compared with the high bitrate range from 20,000 to 40,000 kbps, demonstrating a superior denoising capability. Overall, these results fully validate the optimized image denoising performance of the DRGNet model in post-processing filtering, as well as its exceptional performance and stability in complex video coding scenarios.

### 4.5. Comparative Analysis with PPFF

To further validate the effectiveness of the proposed neural network model in video quality enhancement, particularly in high-resolution video optimization, and to assess its overall performance, we compare it with a recent state-of-the-art method. The experimental results are shown in Table 9 and Table 10. The evaluation was conducted under RA and LDB configurations, following the JVET CTC guidelines, using BD-Rate as the metric for coding efficiency.

**Figure 12:**
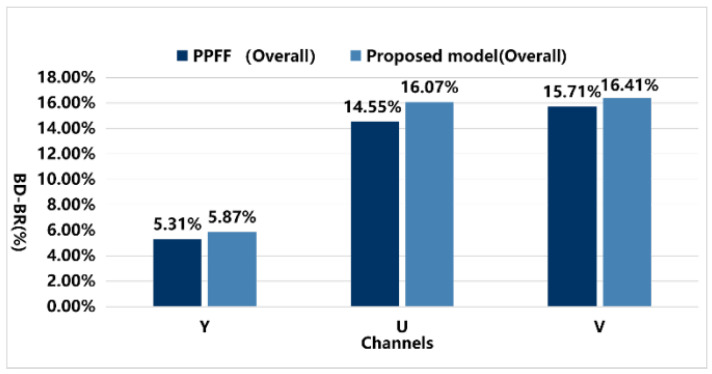
Under the RA configuration, compare the average values of the PPFF model and the proposed model across the A1, A2, B, and C test video datasets.

**Figure 13:**
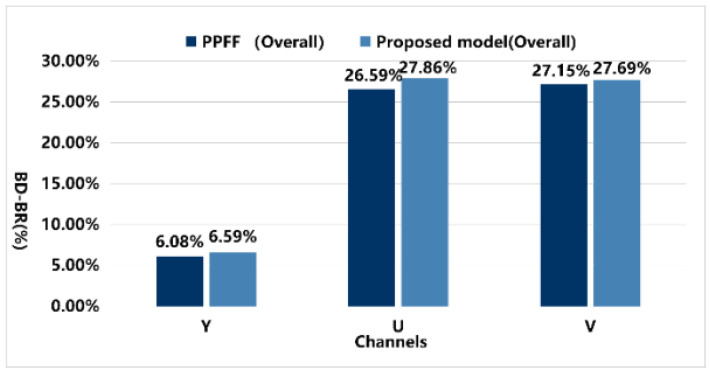
Under the LDB configuration, compare the average values of the PPFF model and the proposed model across the B and C test video datasets.

The results indicate that the DRGNet method achieved BD−Rate values of −5.87% and −6.59% for the Y component in the RA and LDB scenarios, respectively, significantly outperforming the PPFF model, which only achieved BD−Rate values of −5.31% and −6.08%. Figure 12 visually presents the overall average data of the PPFF and DRGNet models under the RA configuration, while Figure 13 shows the average data comparison in the LDB environment.

**Figure 14:**
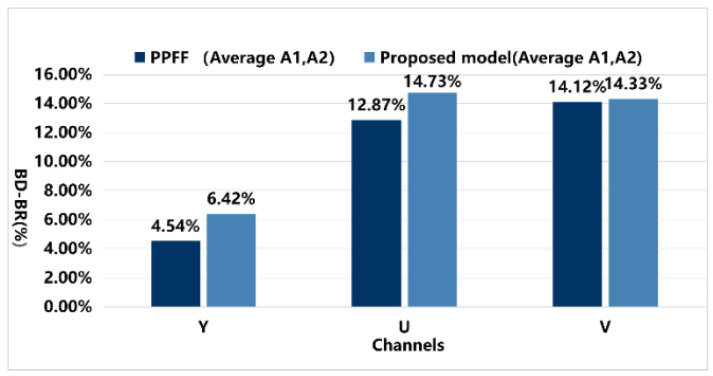
Under RA configuration, compare average performance of the PPFF model and the proposed model on the high-resolution test video datasets.

Notably, in the high-resolution video RA configuration, the A1 and A2 video groups exhibited excellent results in terms of the Y component. Both groups consist of high-resolution videos, aligning closely with the core objective of our experiment. Under this configuration, the proposed model achieved BD−Rate values of −5.55% and −6.30% for the Y component, averaging −6.42%, which is significantly higher than the PPFF model’s −4.38% and −5.30%, averaging −4.84%. This demonstrates remarkable bitrate optimization in high-resolution video scenarios. The growth in Y values is more clearly illustrated in Figure 14.

Moreover, the proposed model consistently showed significant bitrate reduction across all formats in RA, LDB, and AI configurations, whereas the PPFF model only achieved optimization in specific scenarios of certain formats. This highlights the comprehensiveness and superiority of our model, showcasing not only stable performance across various video coding configurations but also delivering substantial quality improvements in high-resolution video processing.

To evaluate the scalability of the proposed model, we analyze its computational cost across multiple input resolutions. As summarized in Table 11, the total number of parameters remains constant at approximately 8.72 million, indicating that the model size does not vary with input resolution. However, the number of floating-point operations (FLOPs) increases significantly with higher spatial resolutions. For instance, at Class A1 and Class A2, the model requires approximately 47.85 × 10^3^ GFLOPs per frame. At Class B, the FLOPs are reduced to around 11.96 × 10^3^, and further drop to 2.30 × 10^3^ GFLOPs for Class C. These results demonstrate the model’s computational scalability and highlight the trade-off between resolution and processing cost during inference.

## 5. Discussion

### 5.1. Key Technical Enhancements

As image quality continues to improve, the demand for high-resolution video has increased rapidly. However, optimizing the quality of high-resolution videos presents greater challenges compared with improving low-resolution videos. Noise in high-resolution videos has a particularly significant impact on fine textures and details. In low-contrast regions, high noise not only makes previously clear scenes blurry or rough but also disrupts the representation of fine textures, thereby affecting the overall visual experience. Therefore, improving the robustness of high-resolution images to noise is crucial. DRGNet, with its innovative design, provides an effective solution to this problem.

In this study, a dual-connected residual gating network module was introduced, with its main function being noise suppression in input features while improving feature representation capabilities through residual connections. The main feature extraction convolution layer is a 2D convolutional layer with a kernel size of 3 × 3, designed to extract primary features from the input. To generate gating features, another 3 × 3 convolutional layer with similar parameters is used. These gating features are passed through a sigmoid activation function to normalize them to the range [0, 1], serving as gating control weights for the main features. The LeakyReLU activation function is applied to process the features, introducing non-linearity. With LeakyReLU, the output is 30% of the input value when the input is less than zero. Allowing the gradient of negative features to flow prevents feature loss, improves gradient propagation, enhances the learning of edge and texture features, and strengthens the network’s training effectiveness and robustness.

The primary objective of this model is to reduce noise in the images, thereby further improving overall image quality. From the experimental results, the effectiveness of this model is clearly validated in video tests with varying resolutions. High-resolution videos typically have more noise points compared with low-resolution videos, such as high-resolution or below, because high-resolution videos capture more details and are more susceptible to sensor noise. In contrast, low-resolution videos, with fewer pixels and limited.

Few models are capable of significantly improving the quality of high-resolution videos while also improving the overall quality of the test set. In fact, enhancing high-resolution videos under the same training conditions is more challenging than improving low-resolution videos. High-resolution videos not only require larger-scale training data but also demand more complex model architectures and solutions to issues such as color distortion and insufficient dynamic range, which are particularly evident in high-resolution scenarios. Through its excellent design, this model successfully overcomes these difficulties and demonstrates significant advantages in improving high-resolution video quality. The main contributions of this study can be summarized as follows:

(I). A CNN-Based Post-Processing Network Design

We propose a single post-processing network based on convolutional neural networks capable of adapting to various encoding scenarios such as RA, AI, and LDB. In this network, we introduce the QP Map quality-aware mechanism to enhance the model’s focus on critical regions while avoiding excessive learning in low-quality areas, thereby improving overall visual quality. QP Map learns to evaluate different regions of an image, enabling the network to precisely distinguish between high-quality areas, such as sharp edges and high-contrast regions, and low-quality areas, such as blurred or noise-contaminated regions. This mechanism ensures that DRGNet prioritizes important image regions rather than applying uniform processing, thereby preserving key details more effectively. Additionally, DRGNet incorporates a noise gating mechanism, where QP Map serves as a noise control parameter to dynamically suppress high-noise areas while preserving essential features. This approach effectively reduces information loss during denoising tasks, minimizing noise interference while accurately retaining image details, significantly enhancing the final output’s visual quality. At DRGNet’s final output stage, the QP Map further optimizes the residual connection computation. By guiding residual learning, it enhances details only in high-quality areas, preventing excessive compensation in low-quality regions and thereby effectively avoiding artifacts. This strategy ensures the precise reconstruction of crucial image details, resulting in a final output that is more natural, sharper, and aligned with high-quality visual presentation standards.

(II). Application of Ultra-Deep Residual Convolutional Neural Networks

We apply DRGNet to post-filtering tasks in video coding and design an efficient image feature extraction module to enhance both visual quality and computational efficiency. The core innovation of DRGNet lies in the introduction of a Gated Path alongside the Main Path, enabling the network to better adapt to inputs of varying quality levels and exhibit greater robustness in feature learning. Specifically, the Gated Path leverages the QP mechanism to dynamically regulate feature flow, effectively suppressing noise while enhancing fine details in key regions. Meanwhile, the Main Path serves as the primary feature extraction module, focusing on global structural modeling and enhancement. The synergy between these two paths significantly improves image enhancement and denoising performance. The introduction of the Gated Path provides a quality-aware dynamic feature modulation mechanism, allowing for more precise noise suppression and finer detail enhancement in critical regions. At the same time, the combination of the Main Path and Gated Path forms a more adaptive feature extraction structure, enabling the network to effectively handle images with varying quality levels. In enhancing the visual quality of high-resolution videos, DRGNet consistently delivers superior results. Additionally, DRGNet integrates 3 × 3 convolution, LeakyReLU activation, and a sigmoid-based gating mechanism, ensuring stable feature learning and effectively improving the visual quality of the final output. The dual-path architecture of DRGNet, consisting of the Main Path and Gated Path, enhances the robustness and efficiency of post-filtering while achieving more accurate quality perception.

(III). A High-Resolution Video-Oriented Neural Network Architecture

In high-resolution video processing, our proposed neural network model has demonstrated remarkable performance improvements, effectively reducing noise and significantly enhancing visual quality. Since each frame of high-resolution video contains a vast amount of pixel information, artifacts and quantization noise become more pronounced during video encoding, demanding higher fidelity in detail preservation. In contrast, videos with lower pixel density tend to exhibit less noticeable noise after compression or decoding due to inherent detail blurring. This experimental approach aligns closely with our research findings, addressing the long-standing challenges of high-resolution video optimization. The Gated Path, guided by the QP Map, enables quality-aware noise suppression, dynamically adjusting the feature flow to precisely mitigate the impact of high-noise regions. Meanwhile, the Main Path is responsible for deep feature extraction and structural recovery, ensuring that critical details are preserved during denoising while enhancing global consistency. Through the synergy of the Gated Path and Main Path, DRGNet leverages QP Map-based dynamic quality perception, achieving superior performance in artifact removal, denoising, and super-resolution enhancement for high-resolution videos. This effectively improves video clarity and visual experience, offering a robust solution for high-quality video encoding optimization.

(IV). Activation Function Synergy for High-Resolution Video Feature Representation

In the DRGNet model, different activation functions play distinct roles at various stages, working together to significantly enhance the model’s performance and stability in image feature extraction tasks. The sigmoid activation function acts as a “feature gate” in the DPRG module, mapping convolutional layer outputs to the [0, 1] range and dynamically adjusting feature weights in conjunction with the QP Map. In high-quality regions such as edges and high-contrast areas, sigmoid outputs approach 1, allowing more key features to pass through, while in low-quality or noisy regions, outputs approach 0, effectively suppressing irrelevant features. LeakyReLU introduces a negative slope, providing non-linear transformation during the feature extraction stage, preventing gradient vanishing, and keeping neurons “active”. It is particularly effective in low-light image enhancement tasks, extracting more details in dark areas and smoothly capturing information in blurred or low-contrast regions, preventing feature loss. When processing high-resolution videos, LeakyReLU significantly improves feature retention in low-quality areas, avoiding information loss caused by compression. The Tanh activation function used at the output stage, normalizes the output by constraining values within the [−1, 1] range. This not only prevents excessive feature values from causing image distortion but also avoids artifacts resulting from over-enhancement in denoising tasks. By balancing the distribution of positive and negative features, Tanh allows the model to handle transitions between light and dark regions naturally, preserving more details in high-brightness scenes and preventing pixel overexposure. Overall, DRGNet leverages the synergistic effects of multiple activation functions: sigmoid refines feature selection, LeakyReLU extracts more details, and Tanh ensures the natural and harmonious appearance of the final output. This comprehensive approach achieves full-process optimization in feature extraction, dynamic modulation, and smooth output, not only significantly enhancing the model’s performance in various image processing tasks but also ensuring exceptional adaptability and stability across diverse scenarios and input qualities, delivering more natural, detailed, and high-quality visual results.

As shown in Table 12, the proposed method achieves a BD-Rate reduction of −5.87% on the Y component, while WCDANN [55] achieves −2.77%, indicating the superior performance of DRGNet, proposed by us, in enhancing luma information.

### 5.2. Limitations of This Study

Although the proposed DRGNet demonstrates notable performance in denoising and enhancing the quality of high-resolution videos, several limitations remain. First, the model’s generalization ability in ultra-high-resolution scenarios (e.g., 4K and above) has not been fully validated. In particular, under the uneven distribution of training data, the model may show reduced effectiveness in restoring fine structural details. Second, the current network architecture includes multiple feature extraction and gating modules, resulting in relatively high computational complexity. This may hinder its deployment in real-time applications or on resource-constrained devices. Moreover, the present model is primarily optimized for reconstructed frames generated by VVC encoding. Its compatibility and adaptability to other video coding standards, such as HEVC or AV1, still require further investigation. Future work may focus on cross-standard model design, lightweight network architectures, and multi-resolution joint training to improve the practical applicability and generalization capability of the proposed method.

### 5.3. Exploration Opportunities in the Proposed Framework

In practical applications, mainstream video formats typically have resolutions of 1920 × 1080 or lower. Although enhancing the quality of high-resolution videos is considerably more challenging than improving low-resolution content, from a technological development perspective, it is essential to improve video quality across different resolutions to advance video processing techniques. Therefore, optimizing the model to ensure consistently high performance across various video resolutions can significantly enhance the adaptability and robustness of video processing methods while also laying a solid foundation for broader application potential and future development.

In our future study, an adaptive QP Map (frame level or coding tree unit level) will be utilized to analyze the effect of constant rate factor (CRF) and constant bit rate (CBR) mode.

### 5.4. Future Works

In future research, we will build upon the current experimental results to further enhance the visual quality of both high-resolution and low-resolution videos. Based on the experience gained from this study, the focus will be on mitigating the risk of model overfitting and effectively integrating frequency information from diverse image content. These optimizations are expected to significantly improve the model’s adaptability to video content at varying resolutions, thereby achieving more robust and flexible video quality enhancement.

## 6. Conclusions

In this paper, we proposed DRGNet, a dual-path residual gating network that effectively integrates a residual structure with a gating mechanism to address key challenges in deep learning-based video enhancement. This design significantly boosts the performance of convolutional networks in image feature extraction and denoising. The proposed DPRG module efficiently removes noise from post-filtered images in video encoding, leading to substantial improvements in overall visual quality. Notably, DRGNet achieves exceptional performance in high-resolution video scenarios, overcoming the limited progress made by traditional methods in this area. Under identical experimental conditions, the model delivers better results on high-resolution videos than on low-resolution ones, effectively leveraging the higher noise characteristics commonly found in high-resolution content. These results demonstrate DRGNet’s strong capability in addressing the challenges of high-resolution video post-filtering and highlight its potential to significantly enhance video quality in practical applications.

## Figures and Tables

**Table 1:** Comparative summary of representative quality enhancement models on codec, dataset, key contributions, and performance metrics.

Model	Target Codec	Dataset/Scene Type	Key Contribution
STDR [48]	N/A (Rendering only)	Synthetic and real-world dynamic scenes. D-NeRF dataset.	Spatio-temporal decoupling for real-time consistency.
CPGA [49]	HEVC/H.265 (HM16.25)	Compressed videos with coding priors: Motion vectors, reference frames, and residuals (trained/tested on datasets like REDS, Vimeo-90K).	Multi-frame enhancement guided by coding priors for artifact removal.
RFDN [50]	N/A (Single Image Super-Resolution)	Benchmark image SR datasets: DIV2K, Set5, Set14, BSD100, Urban100, Manga109.	Residual distillation for lightweight SR.
MFQE2.0 [51]	HEVC/H.265, AVC/H.264	Compressed video sequences exhibiting quality fluctuations; evaluated on datasets including Xiph.org and JCT-VC	PQF-guided multi-frame enhancement using motion-compensated CNN.
PPFF [52]	H.266/VVC	VVC compressed videos under standard configurations.	The network fuses features from different levels to enhance spatial and semantic representations.
ASQE-CNN [53]	H.266/VVC (VTM reference)	VVC-decoded frames under AI, RA, LDB configurations (e.g., JVET CTC test sets).	Shared-weight attention CNN for low-complexity BD-Rate savings.
CVEGAN [54]	H.266/VVC	VVC decoded video frames using CLIC 2022 test sequences and VTM outputs.	Multi-frame GAN enhancement with BD-Rate gains over VTM.
WCDANN [55]	VVC (VTM-11.0-NNVC)	Multiple classes (A1, A2, B, C, D, E) under RA, AI, LDP.	Lightweight CNN with weakly connected dense attention and depthwise separable convolutions (WCDABs) for efficient feature extraction.

**Table 2:** CNN model selection strategy based on QP.

Model	QP Range
$M_{QP=22}$	QP<24.5
$M_{QP=27}$	24.5≤QP<29.5
$M_{QP=32}$	29.5≤QP<34.5
$M_{QP=37}$	34.5≤QP<39.5
$M_{QP=42}$	QP≥39.5

**Table 3:** Video resolution distribution and usage details of the BVI-DVC training dataset.

Video Resolution	Number of Videos	Frames	Bit Depth	Chroma
3840 × 2160	200	2000	10	4:2:0
1920 × 1080	200	2000	10	4:2:0
960 × 544	200	2000	10	4:2:0
480 × 272	200	2000	10	4:2:0

**Table 4:** Video classes and encoding parameter configuration of the JVET NNVC CTC test set.

Class	Video Resolution	Number of Videos	Frames	Bit Depth	Chroma
A1	3840 × 2160	3	1314	10	4:2:0
A2	3840 × 2160	3	1400	10	4:2:0
B	1920 × 1080	5	2802	10	4:2:0
C	832 × 480	4	1903	10	4:2:0

**Table 5:** The compression performance of the proposed method for RA configuration.

Class	Sequence	Y	U	V
**A1**	Tango	−5.48%	−22.48%	−20.56%
	FoodMarket	−3.82%	−10.52%	−8.76%
	Campfire	−7.35%	−8.16%	−19.42%
	**Average**	−5.55%	−13.72%	−16.25%
**A2**	**CatRobot**	−7.61%	−25.62%	−22.01%
	**DaylightRoad**	−8.75%	−21.50%	−19.51%
	ParkRunning	−2.54%	−2.55%	−2.66%
	**Average**	−6.30%	−16.56%	−14.73%
**B**	MarketPlace	−3.47%	−18.38%	−15.71%
	RitualDance	−6.00%	−16.57%	−17.18%
	Cactus	−5.11%	−13.92%	−11.56%
	BasketballDrive	−6.63%	−19.70%	−23.43%
	BQTerrace	−6.09%	−13.82%	−8.18%
	**Average**	−5.46%	−16.48%	−15.21%
**C**	BasketballDrill	−7.27%	−15.50%	−22.19%
	BQMall	−7.02%	−21.15%	−22.86%
	PartyScene	−5.61%	−10.94%	−8.19%
	RaceHorses	−5.31%	−20.27%	−23.91%
	**Average**	−6.30%	−16.96%	−19.29%
**Overall**		−5.87%	−16.07%	−16.41%

**Table 6:** The compression performance of the proposed method for LDB configuration.

Class	Sequence	Y	U	V
**B**	MarketPlace	−3.50%	−32.08%	−25.54%
	RitualDance	−4.92%	−23.68%	−21.34%
	Cactus	−6.16%	−25.03%	−25.21%
	BasketballDrive	−6.00%	−26.54%	−26.71%
	**BQTerrace**	−8.61%	−32.04%	−24.92%
	**Average**	−5.84%	−27.87%	−24.74%
**C**	BasketballDrill	−7.65%	−26.70%	−32.48%
	**BQMall**	−9.61%	−27.51%	−30.20%
	PartyScene	−6.98%	−27.86%	−26.98%
	RaceHorses	−5.89%	−29.26%	−35.87%
	**Average**	−7.53%	−27.83%	−31.38%
**Overall**		−6.84%	−27.86%	−27.69%

**Table 7:** The compression performance of the proposed method for AI configuration.

Class	Sequence	Y	U	V
**A1**	Tango	−4.88%	−11.85%	−13.09%
	FoodMarket	−4.70%	−9.55%	−7.45%
	Campfire	−5.53%	−4.22%	−11.49%
	**Average**	−5.04%	−8.54%	−10.68%
**A2**	**CatRobot**	−8.59%	−18.14%	−17.73%
	**DaylightRoad**	−9.11%	−16.60%	−13.88%
	ParkRunning	−2.28%	−2.56%	−2.69%
	**Average**	−6.66%	−12.43%	−11.43%
**B**	MarketPlace	−4.06%	−13.79%	−12.18%
	RitualDance	−7.11%	−15.10%	−14.54%
	Cactus	−5.05%	−7.16%	−8.92%
	BasketballDrive	−5.32%	−8.83%	−16.19%
	BQTerrace	−4.42%	−8.29%	−6.71%
	**Average**	−5.19%	−10.64%	−11.71%
**C**	BasketballDrill	−8.55%	−8.59%	−24.58%
	BQMall	−7.71%	−11.62%	−15.43%
	PartyScene	−4.66%	−0.80%	−5.62%
	RaceHorses	−5.07%	−14.39%	−20.55%
	**Average**	−6.50%	−8.85%	−15.32%
**Overall**		−5.80%	−10.10%	−12.41%

**Table 8:** Performance evaluation across VVC test conditions: PSNR improvements on YUV channels.

Test	Sequence Class	ΔPSNR-Y (dB)	ΔPSNR-U (dB)	ΔPSNR-V (dB)
RA	A1	0.153	0.225	0.308
	A2	0.134	0.211	0.235
	B	0.169	0.289	0.360
	C	0.270	0.405	0.592
LDB	B	0.282	0.449	0.582
	C	0.284	0.701	0.887
AI	A1	0.140	0.215	0.248
	A2	0.108	0.251	0.230
	B	0.322	0.324	0.662
	C	0.322	0.324	0.662

**Table 9:** BD-Rate comparison between the proposed method and [52] for RA configuration.

Class	PPFF [52]			Proposed		
	Y	U	V	Y	U	V
A1	−4.38%	−11.31%	−15.58%	−5.55%	−13.72%	−16.25%
A2	−5.30%	−14.42%	−12.65%	−6.30%	−16.56%	−14.73%
**Average (A1, A2)**	−4.84%	−12.87%	−14.12%	−6.42%	−14.73%	−14.33%
B	−5.08%	−15.08%	−14.83%	−5.46%	−16.48%	−15.21%
C	−6.28%	−11.31%	−19.21%	−6.30%	−16.96%	−19.29%
**Overall**	−5.31%	−14.55%	−15.71%	−5.87%	−16.07%	−16.41%

**Table 10:** BD-Rate comparison between the proposed method and the LDB configuration.

Class	PPFF [52]			Proposed		
	Y	U	V	Y	U	V
B	−5.25%	−26.03%	−23.95%	−5.84%	−27.87%	−24.74%
C	−7.12%	−27.29%	−30.35%	−7.53%	−27.83%	−31.38%
**Overall**	−6.08%	−26.59%	−27.15%	−6.59%	−27.86%	−27.69%

**Table 11:** Inference complexity of the proposed model across multiple input resolutions.

Class	Resolution	FLOPs (G)	Total Parameters (M)
A1	3840 × 2160	47,850.78	8.72
A2	3480 × 2160	47,850.78	8.72
B	1920 × 1080	11,961.2	8.72
C	832 × 480	2303.6	8.72

**Table 12:** BD-Rate comparison between the proposed method and WCDANN [55] under RA configuration.

Class	WCDANN [55]			Proposed		
	Y	U	V	Y	U	V
A1	−2.23%	N/A	N/A	−5.55%	−13.72%	−16.25%
A2	−2.70%	N/A	N/A	−6.30%	−16.56%	−14.73%
B	−2.73%	N/A	N/A	−5.46%	−16.48%	−15.21%
C	−3.43%	N/A	N/A	−6.30%	−16.96%	−19.29%
**Overall**	−2.77%	N/A	N/A	−5.87%	−16.07%	−16.41%

## Data Availability

The data presented in this study are openly available in [60].
